# Blended Learning Curriculum for Training Faculty Physicians Performing the Extended Focused Assessment With Sonography in Trauma (eFAST)

**DOI:** 10.7759/cureus.88067

**Published:** 2025-07-16

**Authors:** Megan L Hilbert, Jordan Rupp, Jeremy S Boyd

**Affiliations:** 1 Department of Emergency Medicine, University of Pittsburgh Medical Center, Pittsburgh, USA; 2 Department of Emergency Medicine, Vanderbilt University Medical Center, Nashville, USA; 3 Department of Emergency Medicine, Veterans Affairs Tennessee Valley Healthcare System, Nashville, USA

**Keywords:** extended focused assessment with sonography in trauma, faculty confidence, point-of-care ultrasound (pocus), ultrasound acquisition, ultrasound interpretation

## Abstract

Objective

In 2016, the American College of Emergency Physicians (ACEP) published policy statements regarding the application, credentialing, and maintenance of point-of-care ultrasound (POCUS) skills. While the Accreditation Council for Graduate Medical Education (ACGME) now necessitates inclusion of ultrasound teaching in emergency medicine residency, this was not the case prior to 2012. Faculty who were not trained with a formal residency ultrasound curriculum must now maintain their skills through a practice-based pathway. This project was primarily aimed at creating a blended learning curriculum to refresh faculty clinical skills by increasing confidence in both the acquisition and interpretation of the extended Focused Assessment with Sonography in Trauma (eFAST) exam. Secondary aims included evaluating participant interest in the utilization of this application and assessing attitudes toward this asynchronous curriculum.

Methods

Participating emergency medicine faculty were asked to engage in the curriculum, which comprised both asynchronous online material and a hands-on scanning session with ultrasound-trained faculty over a six-week period. Pre- and post-surveys were administered to gauge physician opinions regarding the utilization of ultrasound in the management of trauma patients and their confidence in acquiring and interpreting standard eFAST exam views. Pre- and post-survey responses were collected, and mean values were calculated and compared via a paired two-tailed t-test with statistical significance established at a p-value < 0.05.

Results

After engaging with the curriculum, there was a statistically significant increase in confidence in obtaining all of the eFAST views and interpretation of all but the right upper quadrant (RUQ) view for all participants. Subanalysis demonstrated that participants greater than 10 years from graduation demonstrated a statistically significant increase in confidence in the acquisition and interpretation of more views as compared to their colleagues who graduated within the last 10 years. The curriculum was well-received, with 91.3% of participants reporting an increased likelihood of utilization of the eFAST exam in the medical management of their trauma patients and a 22.3% reduction in perceived barriers to the use of this exam after interventions of this research study. Finally, 96% of participants would be interested in similar training for other ultrasound applications.

Conclusions

We were able to demonstrate an effective model for increasing faculty confidence in the process of refreshing core ultrasound skills as well as increasing interest in the utilization of ultrasound in the management of trauma patients. This seemed to be reflected more significantly in those who are more than 10 years from graduating from an emergency medicine residency program.

## Introduction

In 2016, the American College of Emergency Physicians (ACEP) published a policy statement regarding the use of point-of-care ultrasound (POCUS) by emergency physicians. It not only designated “core applications” of ultrasound but also discussed practical application, credentialing, and maintenance of skills [1]. While residency training now includes POCUS as an Accreditation Council for Graduate Medical Education (ACGME) qualification for graduation, this was not the case prior to 2012. This means that 28% of the current population of emergency physicians did not have this as a cornerstone to their education [2]. ACEP has recognized this divide and has created a practice-based pathway to allow for ultrasound credentialing. No matter the pathway (residency or practice-based), the practice of clinical ultrasound requires skill maintenance and ongoing education. Namely, providers are recommended to complete a minimum of 25 scans and three hours of continuing education every two years. This study is aimed at not only helping faculty to complete these requirements in a novel way at this institution but also increasing confidence in the acquisition and interpretation of a core POCUS application, the extended Focused Assessment with Sonography in Trauma (eFAST) exam.

From 2000 to 2010, trauma-related deaths in the United States increased by 22.8%. In that same timeframe, there was not a commensurate increase in the US population to explain the increase [3]. More recently, the Centers for Disease Control and Prevention (CDC) Web-based Injury Statistics Query and Reporting System (WISQARS) database shows that unintentional injury was the third leading cause of death in all age groups and was the leading cause of death in those aged one to 44 years old in 2019 [4]. With trends of increasing traumatic injuries presenting to the emergency department (ED), it is incumbent upon emergency providers to quickly recognize life-threatening traumatic pathology and intervene. Advanced Trauma Life support (ATLS) now includes the eFAST exam as a part of this early recognition, including it in the primary survey of patients presenting with trauma.

Academic faculty physicians have a wealth of knowledge, but cognitive gains can be increased in a positive manner through attendance at a simple workshop [5]. Pertaining to ultrasound teaching in particular, studies demonstrate that a more experienced scanner increases the accuracy of image acquisition, particularly eFAST [6]. Studies have demonstrated that ultrasound-naive paramedics can “interpret and perform well on eFAST in a simulation situation with minimal training” [7]. If literature can demonstrate such gains in individuals without prior training, it suggests that those with prior exposure should also be able to do well with minimal continued education.

A recent study published by Schwid and colleagues at Brigham and Women’s Hospital discussed the use of “refresher courses” to increase faculty confidence in nine basic emergency ultrasound applications. They demonstrated that sessions as short as 30 minutes of both didactic and hands-on practice could indeed increase provider perceived confidence, with the most noticeable increase in providers greater than 10 years from residency graduation [8]. Much literature has recently focused on “blended learning” models (e-learning as well as other modalities) for teaching medical students [9]. It has been shown that “web-based ultrasonography and eFAST didactics are comparable to traditional classroom lectures and result in similar knowledge retention” [9]. The study also showed increased satisfaction in the groups that were able to complete computer-based learning modules. A study by Chenkin and colleagues has demonstrated that procedures as complicated as ultrasound-guided vascular access can be well accomplished with a web-based curriculum [10]. These studies suggest that maintenance of ultrasound competency, which includes medical knowledge, procedural skill to acquire images, and clinical aptitude to integrate imaging findings into patient care as defined by the Ultrasound Competency Work Group, could be achieved with a multimodal educational module [11].

Clinical educators have a myriad of topics that vie for their time, including their educational responsibilities as well as their clinical and administrative responsibilities. This can often make it difficult to schedule teaching sessions for continuing medical education as well as to maintain ultrasound credentialing. We aimed to develop an easily accessible, asynchronous, and low time-cost training module that addressed all aspects of eFAST competency. The training module includes an online comprehensive didactic video refresher, asynchronous questions with linked video explanations highlighting key learning points, and hands-on instruction during participants’ clinical shifts. We hypothesized that this blended learning course would result in faculty engagement and increase confidence in the acquisition and interpretation of the eFAST exam.

## Materials and methods

Study design and participants

This study was a prospective analysis of a cohort of board-certified attending emergency physicians who practice at an academic tertiary medical center. The cohort is representative of physicians who are from one to 32 years post-graduation of a certified emergency medicine residency. This intentionally broad cohort represents providers with a wide array of ultrasound backgrounds, both in ultrasound-specific learning and clinical utilization. All participants attested to having received eFAST-specific training in the past; however, the timeframe in which they received this training ranged from one month up to 13 years prior to the study period. This study was submitted to the local institutional review board and received a waiver as a quality improvement project. Participants all volunteered to participate.

Curriculum

The faculty eFAST POCUS curriculum consisted of an online recorded lecture, QuizTime quizzes (QuizTime, Vanderbilt University, Nashville, TN, USA), and hands-on scanning sessions. The approximately seven-minute recorded lecture addressed the utilization of the eFAST exam via clinical scenario and reviewed orientation and positioning of the probe, review of relevant anatomy, discussion of what constitutes pathology, comparison of normal versus pathologic findings, as well as common mistakes and pitfalls. While it addressed a large amount of information, it did assume baseline knowledge, which is to be expected of a practicing emergency medicine provider. The lecture was reviewed for accuracy by two content experts.

**Figure 1:**
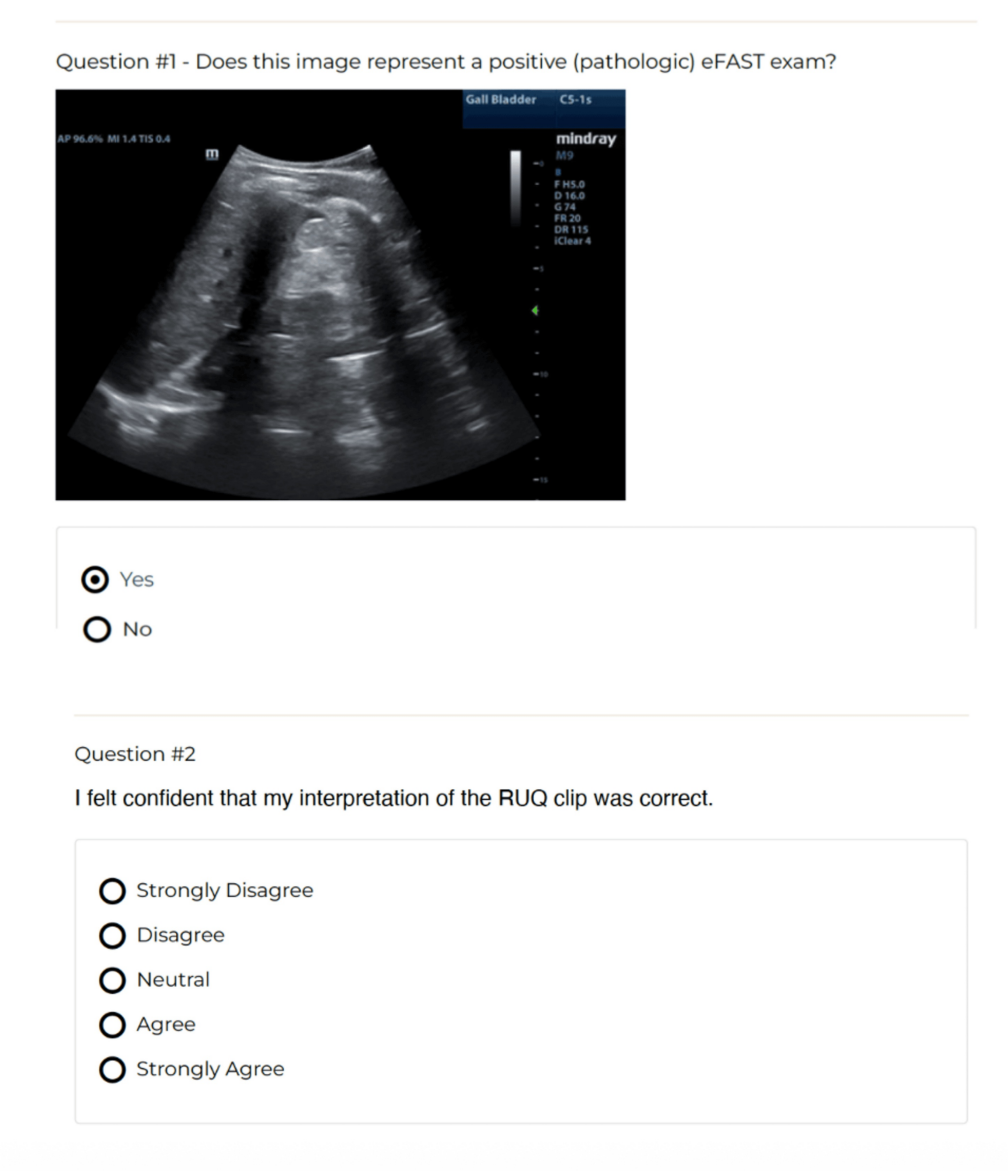
Example of QuizTime question

QuizTime study questions were delivered via email to the participants. Four questions (two content and two confidence) were delivered per week over the course of six weeks, resulting in a total of 24 questions. Content questions contained various eFAST ultrasound clips. The participant was asked to determine whether the eFAST view was “positive” or “negative” for pathologic free fluid and then rate their confidence in this determination. Figure 1 and Video 1 show examples of the QuizTime question and its formatting.

**Video 1:** QuizTime video example RUQ: right upper quadrant Positive RUQ clip

Two questions were formulated for each view (right upper quadrant (RUQ), left upper quadrant (LUQ), subxiphoid, cardiac, and male and female pelvic), respectively. The two questions regarding the same view were not delivered in the same week. Therefore, if participants chose not to engage with the content that week, they were not missing a review of an entire component of the eFAST exam. These videos represented a varying degree of difficulty of interpretation from novice to expert and contained associated explanations to further the asynchronous learning component. After completing the content question, participants were then asked to rate their confidence in their answer. This served as immediate feedback, either supporting a high degree of confidence in those who got the content answer correct or pointing out where a participant might be able to improve. The decision was made to deliver a total of four questions per week to increase the likelihood of completion, not significantly increase the burden on the schedule and time demands of the participants, while also serving as a reminder to the participants to continue to engage with the curriculum. Questions remained available for four weeks after delivery. The content was again reviewed for accuracy by two content experts.

Finally, the hands-on session was completed with an ultrasound fellow or fellowship-trained faculty member to ensure appropriate acquisition and interpretation of the eFAST in real time. The great majority of sessions were completed by the ultrasound fellow to keep consistency among this variable. Sessions consisted of the fellow observing at the bedside while the participant completed an eFAST exam, addressing any questions that arose and adjusting the technique as needed to optimize the view. This scanning session was completed during the participating faculty’s clinical shift so as not to increase time demands on the participants. 

Both the recorded lecture and hands-on scanning were available to participants throughout the entire six-week study period. The QuizTime questions were timed to be delivered weekly and were available for four weeks after delivery.

Questionnaire and knowledge assessments

Participants were administered a pre-survey via RedCap software (RedCap, Vanderbilt University, Nashville, TN, USA) to evaluate their base level of confidence in the acquisition and interpretation of the standard views in the eFAST examination. For the purposes of comparing pre- and post-test surveys, individuals self-assigned an identification number that they were asked to remember over the course of the study period. QuizTime questions were emailed to the participants on a weekly basis for completion during the study period. At the end of the study period, participants were emailed a link to complete a post-survey to evaluate the efficacy of the interventions in increasing their confidence in both the acquisition and interpretation of the eFAST exam.

Data analysis

Pre- and post-survey questionnaire responses were measured with a Likert scale, with a Likert scale of one defined as “not confident at all” and a Likert scale of five defined as “extremely confident.” An increase in mean Likert scale rating represented an increase in confidence. Pre- and post-intervention mean values were then compared using a paired two-tailed t-test with statistical significance established at a p-value < 0.05. The data set was then subanalyzed by time from graduation (those who graduated within the last 10 years versus those who graduated more than 10 years ago). An attempt was made to include all participant data; however, some portions of the surveys were left unanswered, and some participants did not recall their self-identifying number, causing there to be an incomplete data set. 

A total of 31 participants (representing 39% of faculty physicians at our site) were enrolled in this program, with one subsequently dropping out due to technical difficulties after completion of the pre-test survey. Participation was voluntary, and recruitment was completed by emailing all academic emergency medicine faculty at the participating site. There were no external incentives offered for participation. Participant demographics are reported in Table 1.

**Table 1:** Participant demographics eFAST: extended Focused Assessment with Sonography in Trauma (eFAST); POCUS: point-of-care ultrasound Breakdown of participant demographics and characteristics highlighting the extent of prior experience with both the acquisition and interpretation of the eFAST exam. The majority of providers have received prior training regarding the eFAST exam and have completed at least 50 prior exams

Characteristic	Number (%)
-	n = 24*
Gender	-
Female	8 (33.3%)
Male	16 (66.7%)
Prior POCUS experience: how many eFAST exams performed?	
0	0 (0%)
1-5	0 (0%)
6-10	1 (4.2%)
11-25	0 (0%)
26-50	1 (4.2%)
51-100	2 (8.3%)
>100	20 (83.3%)
Prior POCUS experience: how many eFAST exams interpreted?	
0	0 (0%)
1-5	0 (0%)
6-10	0 (0%)
11-25	1 (4.2%)
26-50	0 (0%)
51-100	5 (20%)
>100	18 (75%)
Prior POCUS eFAST learning	
Yes	22 (91.7%)
No	2 (8.3%)

**Figure 2:**
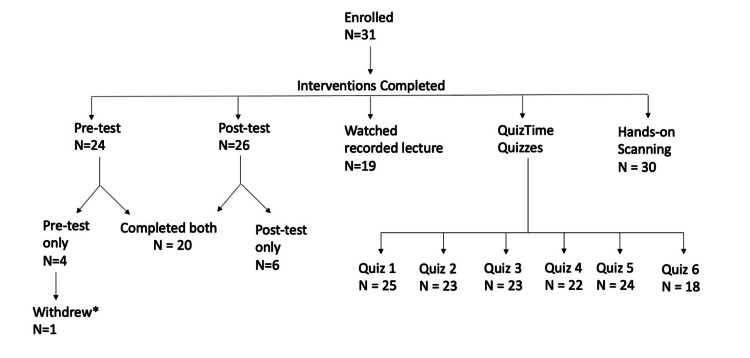
Breakdown of completion of portions of the curriculum *Participant withdrew due to technical difficulties in accessing portions of the curriculum While 31 participants were enrolled in this project originally, there was attrition with regard to completion of various portions of the curriculum. This highlights potential inherent challenges with the implementation of an asynchronous curriculum

A complete review of participant engagement and completion of portions of the curriculum is reported in Figure 2.

## Results

The primary measured outcome was a change in confidence of faculty physicians with regard to image acquisition and interpretation of the eFAST exam views.

eFAST acquisition

In terms of confidence in image acquisition pre-intervention, the mean confidence of obtaining views was the lowest for the subxiphoid and pulmonary view when all participants were evaluated. The remaining views had a pre-intervention mean confidence value greater than or equal to four on the Likert scale. Post-intervention, all mean confidence values were found to have increased from pre-intervention means, and all views experienced a statistically significant increase in confidence post-intervention when evaluated via a two-tailed paired t-test (Table 2).

**Table 2:** Confidence in obtaining the eFAST views-all data eFAST: extended Focused Assessment with Sonography in Trauma; RUQ: right upper quadrant; LUQ: left upper quadrant When considering all participant data, mean confidence increased overall across all views, and all were statistically significant in this increase

View	Relation to intervention	1 = not confident at all	2	3	4	5 = extremely confident	Number (n)	Mean	Standard deviation	95% CI	P two-tail	Degrees of freedom (df)	T statistic
RUQ	Pre	0 (0%)	0 (0%)	1 (4.2%)	11 (45.8%)	12 (50%)	24	4.458	0.5882	4.223-4.693	-	-	-
-	Post	0 (0%)	0 (0%)	0 (0%)	8 (33.3%)	16 (66.7%)	24	4.667	0.4815	4.474-4.860	0.0218	23	-2.46
LUQ	Pre	0 (0%)	2 (9.1%)	1 (4.5%)	10 (45.5%)	9 (40.9%)	22	4.182	0.9069	3.803-4.561	-	-	-
-	Post	0 (0%)	1 (4.0%)	3 (12%)	6 (24%)	15 (60%)	25	4.4	0.866	4.061-4.739	0.0106	21	-2.806
Male pelvic	Pre	0 (0%)	1 (4.3%)	3 (13.0%)	3 (34.7%)	11 (47.8%)	18	4.333	0.9701	3.885-4.781	-	-	-
-	Post	0 (0%)	0 (0%)	0 (0%)	6 (25%)	18 (75%)	24	4.75	0.4423	4.573-4.927	0.0081	17	-3
Female pelvic	Pre	0 (0%)	1 (4.3%)	7 (30.4%)	6 (26.1%)	9 (39.1%)	23	4	0.9535	3.610-4.390	-	-	-
-	Post	0 (0%)	0 (0%)	1 (4.2%)	10 (41.6%)	13 (54.2%)	24	4.5	0.5898	4.264-4.736	0.0002	22	-4.491
Subxiphoid	Pre	0 (0%)	3 (13.0%)	4 (17.4%)	10 (43.5%)	6 (26.1%)	23	3.826	0.984	3.424-4.228	-	-	-
-	Post	0 (0%)	0 (0%)	2 (8.7%)	8 (34.8%)	13 (56.5%)	23	4.478	0.6653	4.206-4.750	0.00002	22	-5.461
Pulmonary	Pre	0 (0%)	2 (10%)	5 (25%)	5 (25%)	8 (40%)	20	3.95	1.0501	3.490-4.410	-	-	-
-	Post	0 (0%)	1 (4.3%)	1 (4.3%)	9 (39.1%)	12 (52.2%)	23	4.391	0.7827	4.071-4.711	0.0016	19	-3.684

If the participants were grouped by time since residency graduation, those more than 10 years from graduation had an increase in mean confidence value in obtaining all views except for the LUQ view (which remained unchanged) post-intervention (Table 3). The male pelvic, female pelvic, subxiphoid, and pulmonary views reached statistical significance for confidence increase.

**Table 3:** Confidence in obtaining the following views-participants > 10 years since graduation from residency RUQ: right upper quadrant; LUQ: left upper quadrant Those with more than 10 years since graduation increased confidence in obtaining all views except the LUQ view. A statistically significant increase was seen in the male pelvic, female pelvic, subxiphoid, and pulmonary views

View	Relation to intervention	1 = not confident at all	2	3	4	5 = extremely confident	Number (n)	Mean	Standard deviation	95% CI	P two-tail	Degrees of freedom (df)	T statistic
RUQ	Pre	0 (0%)	0 (0%)	1 (11.1%)	5 (55.6%)	3 (33.3%)	9	4.222	0.667	3.786-4.658	-	-	-
-	Post	0 (0%)	0 (0%)	0 (0%)	4 (50%)	4 (50%)	8	4.5	0.5345	4.130-4.870	0.0796	7	-2.049
LUQ	Pre	0 (0%)	1 (12.5%)	0 (0%)	4 (50%)	3 (37.5%)	8	4.125	0.991	3.438-4.812	-	-	-
-	Post	0 (0%)	0 (0%)	2 (25%)	3 (37.5%)	3 (37.5%)	8	4.125	0.8345	3.547-4.703	1	7	0
Male pelvic	Pre	0 (0%)	1 (11.1%)	3 (33.3%)	3 (33.3%)	2 (22.2%)	9	3.667	1	3.014-4.320	-	-	-
-	Post	0 (0%)	0 (0%)	0 (0%)	4 (50%)	4 (50%)	8	4.5	0.5345	4.130-4.870	0.0011	7	-5.292
Female pelvic	Pre	0 (0%)	1 (11.1%)	4 (44.4%)	3 (33.3%)	1 (11.1%)	9	3.444	0.8819	2.868-4.020	-	-	-
-	Post	0 (0%)	0 (0%)	1 (12.5%)	5 (62.5%)	2 (25%)	8	4.125	0.6409	3.681-4.569	0.0002	7	-7
Subxiphoid	Pre	0 (0%)	3 (37.5%)	2 (25%)	3 (37.5%)	0 (0%)	8	3	0.9258	2.358-3.642	-	-	-
-	Post	0 (0%)	0 (0%)	0 (0%)	3 (42.9%)	4 (57.1%)	7	4.571	0.5345	4.175-4.967	0.0001	6	-9.295
Pulmonary	Pre	0 (0%)	1 (12.5%)	3 (37.5%)	3 (37.5%)	1 (12.5%)	8	3.5	0.9258	2.858-4.142	-	-	-
-	Post	0 (0%)	0 (0%)	1 (14.3%)	4 (57.1%)	2 (28.6%)	7	4.143	0.6901	3.632-4.654	0.001	6	-6

Those less than 10 years from graduation demonstrated an increase in their mean confidence values for all views when comparing pre- to post-intervention measures, but only the subxiphoid view met statistical significance (Table 4).

**Table 4:** Confidence in obtaining the following views-participants <10 years since graduation of residency RUQ: right upper quadrant; LUQ: left upper quadrant Those less than 10 years from graduation increased mean confidence in obtaining all views, but only the subxiphoid view reached a statistically significant increase. This may indicate that while improvements in confidence can be made for this cohort, with more recent training in the application, there may be a ceiling effect on how much improvement can be made

View	Relation to intervention	1 = not confident at all	2	3	4	5 = extremely confident	Number (n)	Mean	Standard deviation	95% CI	P two-tail	Degrees of freedom (df)	T statistic
RUQ	Pre	0 (0%)	0 (0%)	0 (0%)	3 (25%)	9 (75%)	12	4.75	0.4523	4.494-5.006	-	-	-
-	Post	0 (0%)	0 (0%)	0 (0%)	1 (9.1%)	10 (90.9%)	11	4.909	0.3015	4.731-5.087	0.168	9	-1.5
LUQ	Pre	0 (0%)	0 (0%)	0 (0%)	6 (50%)	6 (50%)	12	4.5	0.5222	4.205-4.795	-	-	-
-	Post	0 (0%)	0 (0%)	1 (9.1%)	1 (9.1%)	9 (81.8%)	11	4.727	0.6467	4.345-5.109	0.368	9	-2.449
Male pelvic	Pre	0 (0%)	0 (0%)	0 (0%)	2 (18.2%)	9 (81.8%)	11	4.818	0.4045	4.579-5.057	-	-	-
-	Post	0 (0%)	0 (0%)	0 (0%)	1 (9.1%)	10 (90.9%)	11	4.909	0.3015	4.731-5.087	0.343	9	-1
Female pelvic	Pre	0 (0%)	0 (0%)	1 (9.1%)	2 (18.2%)	8 (72.7%)	11	4.636	0.6742	4.238-5.034	-	-	-
-	Post	0 (0%)	0 (0%)	0 (0%)	3 (27.3%)	8 (72.7%)	11	4.727	0.4671	4.451-5.003	Undefined	9	Undefined
Subxiphoid	Pre	0 (0%)	0 (0%)	0 (0%)	6 (50%)	6 (50%)	12	4.5	0.5222	4.205-4.795	-	-	-
-	Post	0 (0%)	0 (0%)	1 (9.1%)	1 (9.1%)	9 (81.8%)	11	4.727	0.6467	4.345-5.109	0.0368	9	-2.449
Pulmonary	Pre	0 (0%)	0 (0%)	1 (10%)	2 (20%)	7 (70%)	10	4.6	0.6992	4.167-5.033	-	-	-
-	Post	0 (0%)	0 (0%)	0 (0%)	3 (27.3%)	8 (72.7%)	11	4.727	0.4671	4.451-5.003	0.347	8	-1

eFAST Interpretation

With regard to the interpretation of the eFAST exam, when considering all participants, the mean confidence of obtaining views pre-intervention was lowest for the female pelvic and pulmonary views. The remaining views had a mean pre-intervention confidence value of four or greater on the Likert scale. Post-intervention, all mean confidence values were found to have increased from pre-intervention means, and these increases were found to be statistically significant for all views but the RUQ view (Table 5).

**Table 5:** Confidence in interpreting the eFAST views-all data eFAST: extended Focused Assessment with Sonography in Trauma; RUQ: right upper quadrant; LUQ: left upper quadrant When considering all participant data, the mean confidence increased (with statistical significance) in the LUQ, male pelvic, female pelvic, subxiphoid, and pulmonary views. This increase in statistical significance mirrors what was found with regard to all participant data in the realm of image acquisition. Notably, confidence in the interpretation of the RUQ view did not increase significantly, suggesting that participants are already confident in interpreting this view and did not require further education with regard to this

View	Relation to intervention	1 = not confident at all	2	3	4	5 = extremely confident	Number (n)	Mean	Standard deviation	95% CI	P two-tail	Degrees of freedom (df)	T statistic
RUQ	Pre	0 (0%)	0 (0%)	1 (4.3%)	11 (47.8%)	11 (47.8%)	23	4.435	0.5898	4.194-4.676	-	-	-
-	Post	0 (0%)	0 (0%)	1 (4.2%)	8 (33.3%)	15 (62.5%)	24	4.583	0.5836	4.350-4.816	0.0829	21	-1.821
LUQ	Pre	0 (0%)	1 (4.5%)	2 (9.1%)	9 (40.9%)	10 (45.5%)	22	4.273	0.827	3.927-4.619	-	-	-
-	Post	0 (0%)	1 (4.2%)	2 (8.3%)	6 (25%)	15 (62.5%)	24	4.458	0.833	4.125-4.791	0.0212	20	-2.5
Male pelvic	Pre	0 (0%)	0 (0%)	4 (18.2%)	11 (50%)	7 (31.8%)	22	4.136	0.7102	3.839-4.433	-	-	-
-	Post	0 (0%)	0 (0%)	1 (4.2%)	9 (37.5%)	14 (58.3%)	24	4.542	0.5882	4.307-4.777	0.0009	20	-3.873
Female pelvic	Pre	0 (0%)	0 (0%)	6 (28.6%)	10 (47.6%)	5 (23.8%)	21	3.952	0.74	3.636-4.268	-	-	-
-	Post	0 (0%)	0 (0%)	1 (4.3%)	11 (47.8%)	11 (47.8%)	23	4.435	0.5898	4.194-4.676	0.0003	19	-4.259
Subxiphoid	Pre	0 (0%)	2 (9.1%)	3 (13.6%)	10 (45.5%)	7 (31.8%)	22	4	0.9258	3.613-4.387	-	-	-
-	Post	0 (0%)	0 (0%)	1 (4.8%)	8 (38.1%)	12 (57.1%)	21	4.524	0.6016	4.267-4.781	0.0007	19	-4.067
Pulmonary	Pre	0 (0%)	3 (15%)	6 (30%)	5 (25%)	6 (30%)	20	3.7	1.0809	3.226-4.174	-	-	-
-	Post	0 (0%)	1 (4.5%)	2 (9.1%)	9 (40.9%)	10 (45.5%)	22	4.273	0.827	3.927-4.619	0.0002	18	-4.609

Participants with more than 10 years from graduation had statistically significant increases in mean confidence of interpreting the female pelvic and pulmonary views (Table 6).

**Table 6:** Confidence in interpreting the following views-participants >10 years since graduation from residency RUQ: right upper quadrant; LUQ: left upper quadrant Those with more than 10 years from graduation demonstrated a statistically significant increase in mean confidence in interpreting the female pelvic and pulmonary views. This cohort increased confidence in the acquisition of four views (male pelvic, female pelvic, subxiphoid, and pulmonary), but only increased confidence in the interpretation of two views (female pelvic and pulmonary)

View	Relation to intervention	1 = not confident at all	2	3	4	5 = extremely confident	Number (n)	Mean	Standard deviation	95% CI	P two-tail	Degrees of freedom (df)	T statistic
RUQ	Pre	0 (0%)	0 (0%)	0 (0%)	5 (62.5%)	3 (37.5%)	8	4.375	0.5175	4.016-4.734	-	-	-
-	Post	0 (0%)	0 (0%)	1 (12.5%)	3 (37.5%)	4 (50%)	8	4.375	0.774	3.859-4.891	0.356	6	-1
LUQ	Pre	0 (0%)	0 (0%)	1 (12.5%)	4 (50%)	3 (37.5%)	8	4.25	0.7071	3.760-4.740	-	-	-
-	Post	0 (0%)	0 (0%)	1 (12.5%)	4 (50%)	3 (37.5%)	8	4.25	0.7071	3.760-4.740	Undefined	6	Undefined
Male pelvic	Pre	0 (0%)	0 (0%)	3 (42.9%)	3 (42.9%)	1 (14.2%)	7	3.714	0.7559	3.154-4.274	-	-	-
-	Post	0 (0%)	0 (0%)	1 (12.5%)	5 (62.5%)	2 (25%)	8	4.125	0.6409	3.681-4.569	0.0756	5	-2.236
Female pelvic	Pre	0 (0%)	0 (0%)	4 (57.1%)	3 (42.9%)	0 (0%)	7	3.429	0.5345	3.033-3.825	-	-	-
-	Post	0 (0%)	0 (0%)	1 (16.6%)	4 (66.8%)	1 (16.6%)	6	4	0.6325	3.494-4.506	0.0161	4	-4
Subxiphoid	Pre	0 (0%)	2 (25%)	1 (12.5%)	5 (62.5%)	0 (0%)	8	3.375	0.9161	2.740-4.010	-	-	-
-	Post	0 (0%)	0 (0%)	0 (0%)	4 (66.7%)	2 (33.3%)	6	4.333	0.5164	3.920-4.746	0.0704	4	-2.449
Pulmonary	Pre	0 (0%)	2 (25%)	4 (50%)	2 (25%)	0 (0%)	8	3	0.7559	2.476-3.524	-	-	-
-	Post	0 (0%)	0 (0%)	2 (28.6%)	4 (57.1%)	1 (14.3%)	7	3.857	0.6901	3.346-4.368	0.025	5	-3.162

Their counterparts (those with less than 10 years from graduation) only met statistical significance for an increase in the mean confidence ratings in the male pelvic view (Table 7).

**Table 7:** Confidence in interpreting the following views-participants <10 years since graduation from residency RUQ: right upper quadrant; LUQ: left upper quadrant Those with less than 10 years from graduation demonstrated mean increases in confidence in interpretation of the views (apart from pulmonary, which was unchanged), but only the male pelvic view reached statistical significance. This suggests that this cohort benefited less from this educational material than the >10 years since graduation cohort (who improved in two views)

View	Relation to intervention	1 = not confident at all	2	3	4	5 = extremely confident	Number (n)	Mean	Standard deviation	95% CI	P two-tail	Degrees of freedom (df)	T statistic
RUQ	Pre	0 (0%)	0 (0%)	0 (0%)	4 (33.3%)	8 (66.7%)	12	4.667	0.4924	4.388-4.946	-	-	-
-	Post	0 (0%)	0 (0%)	0 (0%)	2 (18.2%)	9 (81.8%)	11	4.818	0.4045	4.579-5.057	0.1679	9	-1.5
LUQ	Pre	0 (0%)	0 (0%)	0 (0%)	5 (41.7%)	7 (58.3%)	12	4.583	0.5149	4.292-4.874	-	-	-
-	Post	0 (0%)	0 (0%)	1 (9.1%)	2 (18.2%)	8 (72.7%)	11	4.636	0.6742	4.238-5.034	0.1679	9	-1.5
Male pelvic	Pre	0 (0%)	0 (0%)	0 (0%)	6 (50%)	6 (50%)	12	4.5	0.5222	4.205-4.795	-	-	-
-	Post	0 (0%)	0 (0%)	0 (0%)	2 (18.2%)	9 (81.8%)	11	4.818	0.4045	4.579-5.057	0.03679	9	-2.449
Female pelvic	Pre	0 (0%)	0 (0%)	0 (0%)	6 (54.5%)	5 (45.5%)	11	4.455	0.5222	4.146-4.764	-	-	-
-	Post	0 (0%)	0 (0%)	0 (0%)	4 (36.4%)	7 (63.6%)	11	4.636	0.5045	4.338-4.934	0.1679	9	-1.5
Subxiphoid	Pre	0 (0%)	0 (0%)	0 (0%)	4 (36.4%)	7 (63.6%)	11	4.636	0.5045	4.338-4.934	-	-	-
-	Post	0 (0%)	0 (0%)	0 (0%)	3 (30%)	7 (70%)	10	4.7	0.483	4.401-4.999	0.3466	8	-1
Pulmonary	Pre	0 (0%)	0 (0%)	1 (10%)	3 (30%)	6 (60%)	10	4.5	0.7071	4.062-4.938	-	-	-
-	Post	0 (0%)	0 (0%)	1 (10%)	3 (30%)	6 (60%)	10	4.5	0.7071	4.062-4.938	Undefined	8	Undefined

Before the completion of this study, attitudes toward utilization of the eFAST exam demonstrated that all participants viewed this ultrasound application as at least “relevant, but not essential” (corresponding to a Likert scale of three or higher) in the clinical management of trauma patients (Table 8).

**Table 8:** Approach to ultrasound use in trauma patient management-all data eFAST: extended Focused Assessment with Sonography in Trauma Overview of participant attitudes with regard to the intervention. This table demonstrates an overall trend toward viewing ultrasound as relevant (if not essential) to the management of these patients post-intervention. There was also a decrease in perceived barriers to the use of this modality after intervention

How essential do you view the eFAST exam to be in the management of your trauma patients?	1 = not relevant	2	3 = relevant, but not essential	4	5 = essential	Number (n)
Pre	0 (0%)	0 (0%)	7 (30.4%)	11 (47.8%)	5 (21.7%)	23
Post	0 (0%)	0 (0%)	4 (16%)	12 (48%)	9 (36%)	25
How interested are you in increasing your utilization of the eFAST exam and billing for this ultrasound study?	1 = Not interested at all	2	3 = Neutral	4	5 = Extremely interested	-
Pre	0 (0%)	1 (4.8%)	5 (23.8%)	8 (38.1%)	7 (33.3%)	21
Post	0 (0%)	0 (0%)	3 (13%)	11 (47.8%)	9 (39.1%)	23
Do you have current barriers that preclude you from instituting ultrasound into your practice?	Yes	No	-	-	-	-
Pre	14 (58.3%)	10 (41.7%)	-	-	-	24
Post	9 (36%)	16 (64%)	-	-	-	25
Reason for perceived barriers-related to	Documentation	Obtaining views	Interpreting images	Integrating into clinical care	-	-
Pre	8 (57.1%)	3 (21.4%)	3 21.4%)	0 (0%)	-	14
Post	3 (33.3%)	1 (11.1%)	4 (44.4%)	1 (11.1%)	-	9

A total of 91.3% of the participants also responded favorably when asked if they were interested in increasing utilization of the eFAST exam. There was an increase in the opinion of how essential the eFAST ultrasound application was, with a 14.5% increase in interest as compared to pre-intervention.

When asked initially, 58.3% of all participants acknowledged barriers existed that precluded the use of ultrasound more regularly in their practice (Table 8). The majority of this was representative of documentation and workflow issues, which were not directly evaluated during this study nor mentioned in the curriculum itself. After intervention, the percentage of providers who acknowledged barriers decreased to 36% (Table 8). There was also a demonstrated shift in the cause of perceived barriers, with post-intervention individuals identifying difficulty in the interpretation of images as the greatest barrier. This shift points to a potential increase in insight and acknowledgement of knowledge gaps in the completion of the eFAST exam. Finally, 96% of the participants would be interested in similar training for other ultrasound applications.

## Discussion

This blended learning curriculum (combining asynchronous material with hands-on coaching by ultrasound faculty) was utilized to increase confidence in both the acquisition and interpretation of the core ultrasound application of eFAST and followed the multimodal educational module suggested by the Ultrasound Competency Work Group [11]. Our initial study focused specifically on the eFAST exam as the study institution is a level one trauma center with over 3,500 annual trauma team activations. Anecdotally, bedside physician evaluation with the eFAST exam at our institution has contributed to enhanced trauma care by enabling more timely escalation of trauma levels and mobilization of life-saving hospital resources.

This curriculum assists with continued medical education requirements and maintenance of ultrasound credentialing by emergency medicine faculty. It demonstrates a degree of flexibility, given that participants completed the majority of the learning on their own time, with the hands-on scanning sessions completed when they were already on a clinical shift. The web-based curricular component was well-received by the participating faculty, similar to the trend seen in literature published by Platz and colleagues [9]. 

The goal of increasing faculty confidence in both image acquisition and interpretation of the eFAST exam was targeted to empower providers to increase their ultrasound utilization in the clinical management of their patients, with our study demonstrating that confidence was most increased in faculty who never had ultrasound training during their own residency training. This follows the trend that was seen in a similar study completed by Schwid and colleagues and highlights the importance of continuing education and renewal of skills of emergency medicine providers (particularly those who did not receive ultrasound training as a part of their residency) [8]. 

It serves to reason that when faculty are more confident in their own ultrasound skills, they are, in turn, more likely to assist in educating emergency medicine residents; this could serve to improve the education of emerging emergency providers who are entering the workforce. Our study did not demonstrate a decrease in confidence for any of the views after engagement with the curriculum and showed a trend toward increasing interest in the utilization of ultrasound clinically. Given the positive response to the interventions of this study, this curriculum might serve as a framework for developing future faculty education in other core ultrasound applications beyond eFAST (Table 9). While the initial formulation of the curriculum is time-intensive and could require administrative buy-in to create, it would not require a large amount of continued administrative work once applied (apart from monitoring for completion). Employing this type of curriculum for one ultrasound application could highlight pitfalls or site-specific adjustments that could be made for better efficacy of future iterations. This same curriculum approach could be applied widely to emergency medicine topics, allowing for generalizability.

**Table 9:** Post-intervention participant opinions eFAST: extended Focused Assessment with Sonography in Trauma; POCUS: point-of-care ultrasound

-	1 = poorly thought out, too long, and not helpful in my clinical practice	2	3 = contained the necessary information, but could be improved and the length could be optimized (either shorter or longer)	4	5 = time-efficient, high yield, and portrayed the correct amount of information	Number (n)
How would you rate the quality of the recorded lecture and hands-on scanning at increasing your knowledge of eFAST?	0 (0%)	1 (4.3%)	0 (0%)	9 (39.1%)	13 (56.5%)	23
-	1 = extremely unlikely	2	3 = neutral	4	5 = extremely likely	-
After this course, how likely are you to increase your utilization of the eFAST exam in the medical management of your trauma patients?	0 (0%)	0 (0%)	2 (8.7%)	13 (56.5%)	8 (34.8%)	23
After this course, how likely are you to increase documentation of the eFAST exam in your trauma patients?	0 (0%)	0 (0%)	3 (13%)	11 (47.8%)	9 (39.1%)	23
-	Yes	No	-	-	-	-
Do you feel that the barriers to your use of the eFAST exam/POCUS were lessened by the interventions of this research study?	21 (84%)	4 (16%)	-	-	-	25
Would you be interested in similar training for other ultrasound applications?	24 (96%)	1 (4%)	-	-	-	25

Future directions could be aimed at evaluating participant competency in the acquisition and interpretation of the eFAST exam by adding a hands-on skill assessment or knowledge-based assessment with a validated competency tool. Conclusions could also be further evaluated in the context of patient outcomes such as time to completion of additional advanced imaging or definitive management of injuries after completion of the eFAST exam, ED length of stay, and the percentage of unlevelled traumas that are then activated after completion of the eFAST exam.

Limitations

Much of the current literature focuses on individuals (at various levels of training) who have had no prior POCUS teaching, in order to attribute all changes in competence and confidence to the intervention they applied. Our research is unique in that participants were emergency medicine faculty who have had varied levels of ultrasound training in the past. It is therefore difficult for us to attribute all reported changes in confidence to the implemented curriculum, as our interventions may instead serve as a reminder of prior knowledge gained. Despite this, the greater reported increases in confidence of individuals who did not complete ultrasound training as a part of their residency (as compared to those who did) suggest that there is a component of new knowledge gained as opposed to just a renewal of old information. There is also a component of selection bias, given that participants were allowed to self-enroll in the study. This suggests that the study population may have more interest in learning POCUS and may not be representative of the wider emergency medicine faculty population. This is further supported by the fact that the majority of participants identified as having completed and interpreted greater than 100 prior eFAST exams, as seen in the pre-intervention survey.

This study was written and planned to account for the clinical duties and busy schedules of the participants and, therefore, was designed as a concise asynchronous intervention. Our study also utilized computer-based educational modules to allow participants to engage with the subject matter at their preferred time; this ultimately resulted in some of the participants not completing portions of the curriculum. Missing participant completion of the educational program, as well as portions of the pre- and post-survey, resulted in gaps within the data, which could sway the calculation of means. While the hands-on component was intended to lessen the time burdens on participants themselves, it was time-intensive for the ultrasound faculty. Scheduling the sessions was completed by the ultrasound fellow, manually contacting all participants. The sessions themselves were completed by the ultrasound faculty presenting to the ED, extracurricular to their own clinical workload, and represented a total of 23 hours of active scanning time. This is an overall large time commitment on the ultrasound faculty and a significant limitation in future implementation. 

With regard to participant views regarding the curriculum, it is possible that the study was not found to be particularly useful, given the fact that this study was completed at an academic center with a highly trained Trauma service. The capabilities of this facility tend to allow for stabilization of a patient (even those with a positive eFAST) in order to complete CT scans to help with surgical planning. As such, the eFAST does not always result in a change in clinical management. The same would likely occur at other academic institutions. Where this could truly affect meaningful change is in the community setting, where a trauma service and additional resources are not readily available.

Finally, the verbiage of the surveys (particularly the post-intervention survey) created compound questions and therefore did not adequately assess individual points. This created ambiguity in the conclusions that could be drawn from this data, as there were two variables in the question stem and only one answer provided. Unfortunately, this limitation was identified after completion of the study, at which point there was no ability to clarify answers with the participants.

## Conclusions

Through the implementation of this curriculum, we were able to demonstrate an effective model for increasing faculty confidence in the process of refreshing core ultrasound skills. There were more statistically significant increases in confidence in faculty who graduated from residency more than 10 years ago and therefore did not receive a formalized ultrasound curriculum as a part of their residency training. This could serve as an effective template for future curriculum development with the goal of increasing faculty confidence for a wide variety of learners and ultrasound backgrounds. In turn, this could assist with maintaining ultrasound competency in different ultrasound applications (although the determination of competency is beyond the scope of this paper). We also demonstrated an increased interest in the utilization of ultrasound in the management of trauma patients, which may help to more quickly identify life-threatening pathology in this patient population and therefore prompt meaningful clinical care.

## References

[1] American College of Emergency Physicians (2017). Ultrasound guidelines: emergency, point-of-care, and clinical utrasound guidelines in medicine. Ann Emerg Med.

[2] Bennett CL, Sullivan AF, Ginde AA, Rogers J, Espinola JA, Clay CE, Camargo CA Jr (2020). National study of the emergency physician workforce, 2020. Ann Emerg Med.

[3] Rhee P, Joseph B, Pandit V, Aziz H, Vercruysse G, Kulvatunyou N, Friese RS (2014). Increasing trauma deaths in the United States. Ann Surg.

[4] (2021). WISQARS: leading causes of death visualization tool. https://wisqars.cdc.gov/data/lcd/home.

[5] Ghazvini K, Mohammadi A, Mohamad J (2014). The impact of the faculty development workshop on educational research abilities of faculties in mashhad university of medical sciences. Future Med Educ J.

[6] Khosravian K, Boniface K, Dearing E, Drake A, Ogle K, Pyle M, Frasure SE (2021). eFAST exam errors at a level 1 trauma center: a retrospective cohort study. Am J Emerg Med.

[7] Buaprasert P, Sri-On J, Sukhuntee J (2021). Diagnostic accuracy of extended focused assessment with sonography for trauma performed by paramedic students: a simulation-based pilot study. Open Access Emerg Med.

[8] Schwid M, Harris O, Landry A, Eyre A, Henwood P, Kimberly H (2019). Use of a refresher course increases confidence in point-of-care ultrasound skills in emergency medicine faculty. Cureus.

[9] Platz E, Goldflam K, Mennicke M, Parisini E, Christ M, Hohenstein C (2010). Comparison of web-versus classroom-based basic ultrasonographic and EFAST training in 2 European hospitals. Ann Emerg Med.

[10] Chenkin J, Lee S, Huynh T, Bandiera G (2008). Procedures can be learned on the web: a randomized study of ultrasound-guided vascular access training. Acad Emerg Med.

[11] Damewood SC, Leo M, Bailitz J, Gottlieb M, Liu R, Hoffmann B, Gaspari RJ (2020). Tools for measuring clinical ultrasound competency: recommendations from the ultrasound competency work group. AEM Educ Train.

